# Highly Efficient NMR Assignment of Intrinsically Disordered Proteins: Application to B- and T Cell Receptor Domains

**DOI:** 10.1371/journal.pone.0062947

**Published:** 2013-05-07

**Authors:** Linnéa Isaksson, Maxim Mayzel, Maria Saline, Anders Pedersen, Joakim Rosenlöw, Bernhard Brutscher, B. Göran Karlsson, Vladislav Y. Orekhov

**Affiliations:** 1 Swedish NMR Centre, University of Gothenburg, Gothenburg, Sweden; 2 Institut de Biologie Structurale, Université Grenoble CEA CNRS, Grenoble, France; University of Alberta, Canada

## Abstract

We present an integrated approach for efficient characterization of intrinsically disordered proteins. Batch cell-free expression, fast data acquisition, automated analysis, and statistical validation with data resampling have been combined for achieving cost-effective protein expression, and rapid automated backbone assignment. The new methodology is applied for characterization of five cytosolic domains from T- and B-cell receptors in solution.

## Introduction

The classical paradigm that protein functionality requires a pre-formed three-dimensional structure has significantly eroded over the last decade under mounting evidence regarding the role of intrinsically disordered proteins (IDPs), inherently devoid of a defined three-dimensional structure [1]–[4]. Bioinformatic analysis and numerous experimental observations indicate that 25–30% of the mammalian proteome is predominantly disordered. The IDPs are particularly abundant in regulation pathways and play a major role in signal transduction. 70% of the proteins involved in signaling have long disordered stretches. NMR spectroscopy is the primary experimental technique for characterizing structural ensembles and transient interactions between IDPs and their partner molecules in solution [5]. However, the traditional biomolecular NMR toolbox, which has been developed and fine-tuned over the past three decades for studying globular proteins, requires adaptation for IDPs. Molecular size is often considered as the main challenge in NMR protein studies. There are two major phenomena that contribute to this. First, signal overlap and complexity of data analysis increases as more and more peaks appear in the spectra. Second, signals in the spectra become broader and sensitivity decreases as spin relaxation becomes faster with slower Brownian tumbling of larger molecules. The two factors, however, have strikingly different effects on structured and highly disordered proteins. Globular proteins usually show good signal dispersion and a low degree of signal overlap, but suffer the most from spin relaxation. NMR studies of intrinsically disordered proteins, on the other side, mainly suffer from inherently low signal dispersion, resulting in server signal overlap. For large protein systems, remarkable progress has been made over the last years in dealing with the relaxation losses [6]–[8], while for disordered proteins, the main efforts were focused on reducing signal overlap [9], [10] taking advantage of the favorable relaxation properties of IDPs. The function of IDPs is related to interactions with a multitude of partner molecules and a response to subtle changes in the solution environment. The new NMR methodology has to focus on elucidation of protein residual structure, transient interactions and minor but functionally important states. Characterization of a disordered protein typically requires preparation and analysis of many protein samples including those with site-specific mutations [11], attached paramagnetic probes [12], variable patterns of selective isotope labeling, different solution conditions, presence of various ligands, etc. Thus, NMR spectroscopy tailored for IDP studies must rely on fast and effective approaches for sample preparation, data acquisition, analysis and statistical validation of the achieved result.

Here we demonstrate for the first time the systematic use of cell-free protein synthesis (CFPS) [13] for producing IDPs for NMR analysis and introduce an integrated approach featuring rapid and low-cost CFPS, fast-pulsing NMR spectroscopy [14] combined with non-uniform data sampling (NUS) and targeted acquisition (TA) [15], automated signal detection and backbone assignment, and a novel statistical validation of the results. The approach is demonstrated on five cytosolic domains of the T- and B-cell receptors (TCR and BCR). Upon ligand binding to the receptors, the immunoreceptor tyrosine-based activation motifs (ITAM) of the cytosolic domains are phosphorylated, which starts the downstream intracellular signaling cascade. The disordered state of the ITAM containing domains in solution has been established previously using the predictions from the amino acid sequence and circular dichroism (CD) data [16], [17]. The structural disorder is also in line with the secondary chemical shifts presented in this paper. The molecular mechanisms of signal transduction through the very flexible domains are still largely unknown and represent the subject of active research.

## Results and Discussion

CFPS is an established tool for producing functional protein samples [18]–[23]. In this work we demonstrate for the first time the systematic use of CFPS for producing IDPs for NMR analysis. It has been noted that CFPS is particularly well suited for disordered proteins. High content of disorder was positively correlated with protein yields and solubility for *E. coli*-based batch CFPS [24]. *In vivo*, regions of low-complexity and disorder of overexpressed heterologous proteins are often prone to proteolytic degradation, which may decrease the yields or even prohibit protein production. In CFPS, the proteolysis is circumvented by short expression time and by adding protease inhibitors directly into the reaction [24]. That no complex folding is needed and that IDPs generally do not aggregate also works in favor of CFPS. Since CFPS is capable of producing proteins of molecular weights well above 50 kDa [23], the CFPS methodology described in this paper should be applicable to any conceivable IDP.

**Figure 1:**
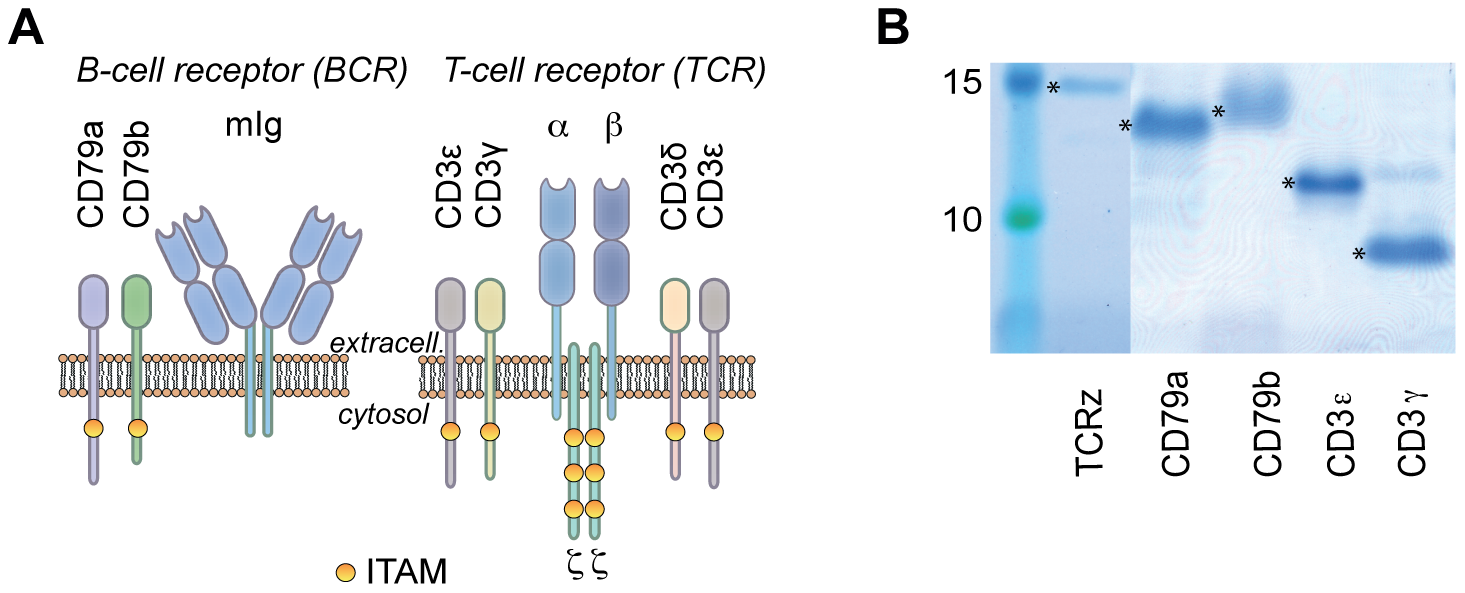
Cell-free expressed cytosolic constructs of the T cell- and B cell receptor. (**A**) Cartoon of the receptors with indicated immunoreceptor tyrosine-based activation motifs (ITAMs), (**B**) SDS-PAGE gel of *in vitro* expressed disordered constructs in levels suitable for NMR experiments.

We use a low-cost, *E. coli*-based batch CFPS system [13]. As shown in Figure 1, five out of six (CD3ε, Cd3γ, TCRζ, CD79a, CD79b) cytosolic domains were successfully expressed and purified with yields sufficient for characterization by NMR. In batch CFPS, the protein synthesis, which is initiated by presentation of the protein gene to the reaction, is mostly finished within one hour. Initial screening of conditions, temperature and different additives can therefore be performed and analyzed very rapidly. For the batch system we use a homemade extract, which only accounts for 4% of the total material cost when producing ^15^N/^13^C labeled protein samples. The most expensive ingredient of the reaction (72% of the costs) is the mixture of double-labeled amino acids (1 mg/mL). With the high level of amino acid incorporation in our CFPS [13], the amount of protein produced in 1 mL of the reaction was sufficient for backbone assignment. This corresponds to a total material cost of about $20 for the NMR sample.

**Figure 2:**
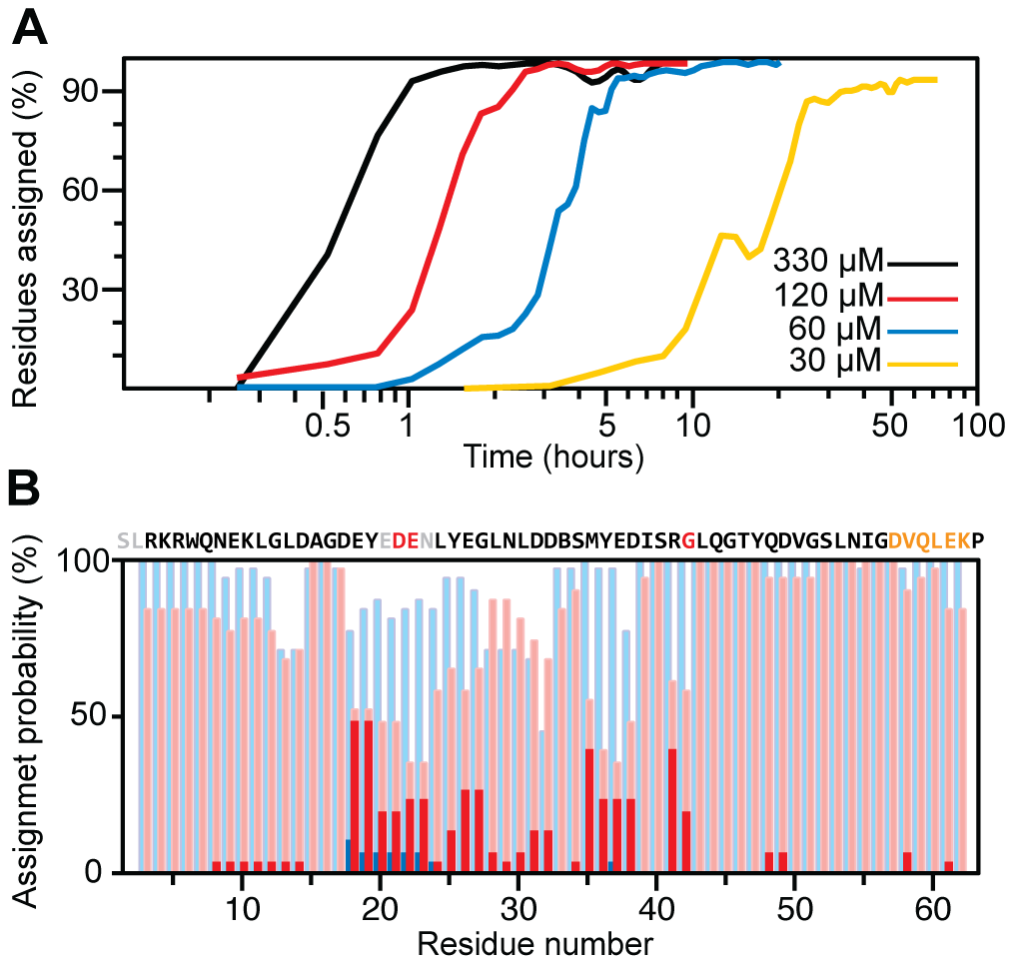
Backbone assignment for CD79a. (**A**) Progress of the assignment during the course of TA procedure for CD79a samples with concentrations of 330, 120, 60, and 30 µM. The horizontal axis shows the total measurement time excluding the HNCO experiment, which was recorded prior to the TA. The spectral processing and automated analysis were always shorter than the data acquisition and, thus, can be performed in real time; (**B**) assignment validation for 60 µM (blue bars) and 30 µM (red bars) samples. The assignment probabilities are plotted for the most probable and alternative assignments (shaded and solid bars, respectively). The probability scores were calculated as a fraction of successful assignments over 30 resample trials.

**Figure 3:**
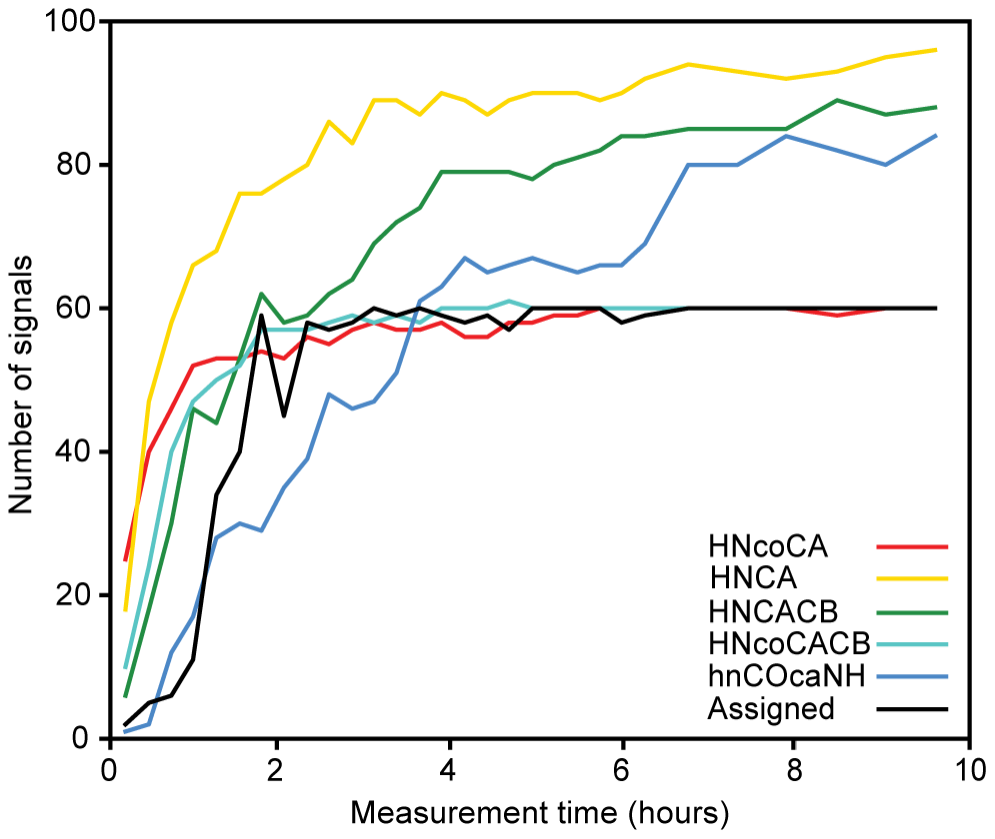
Progress of targeted acquisition versus total measurement time for a 120 µM sample of CD79a. Build-ups are shown for the number of assigned residues and the number of detected peaks in individual BEST-TROSY-type experiments [14].

Chemical shifts report on IDP structure and interactions [5], [11]. For the sequential backbone assignment we use the following TA procedure: (i) a small fraction of data is acquired in NUS mode for each of the triple resonance BEST-TROSY experiments; (ii) the data are combined with the earlier collected measurements (if present) and processed using co-MDD [25]; (iii) signals are detected in the resulting one-dimensional spectral shapes and used for the automated assignment with the AutoAssign software [26]; (iv) scores on completeness of the peak lists and the achieved backbone assignment are evaluated, and, if needed, the procedure is repeated starting from step (i). The TA procedure is finished when the number of peaks in the experiments and number of assigned residues level off and do not increase over several consecutive rounds (Figure 2A and Figure 3).

Figure 2A shows the results of the TA assignment for CD79a samples with concentrations ranging from 30 µM to 330 µM. For the 330 µM sample, which is a typical protein concentration used for triple resonance experiments, the backbone assignment is essentially complete in one hour. This is about hundred times faster in comparison with the conventional methods of data collection and analysis. The result also surpasses our previously reported results for globular proteins and the disordered cytosolic domain of TCRζ [25]. 30 µM is among the lowest protein concentrations for which protein backbone sequential assignment has ever been reported.

Validation of the results of complex analysis such as protein sequential assignment or structure calculation is an important problem [26]–[29]. Peak identification in the spectra containing signal overlap, noise, and artifacts is an intrinsically ill-defined task with ambiguous outcome. In the downstream analysis, uncertainties and errors in peak lists are among the major reasons for wrong or ambiguous solutions. In principle, the best way for estimating the contribution of the spectral noise to the outcome of a complicated non-linear analysis is to repeat the entire analysis on a new set of measurements obtained under the same experimental conditions. Unfortunately, this method is seldom practical. The resampling methods were developed as close proxies for the repeated measurement approach. In this work, we introduce a new method, which uses a jackknife-type resampling 30,31 procedure to cross-validate results of the analysis versus raw spectroscopic data. The method is complementary to the traditional sequential assignment validation tools [26], [28], [32].

In this work, the quality of the obtained assignments and existence of alternative solutions are evaluated with a newly presented data resampling method. The statistics on multiple assignments is obtained by repeating steps (ii–iv) of the TA procedure described above, on a number of data subsets, each containing 70% of the randomly selected points acquired in the triple resonance experiments. This jackknife-type statistical procedure addresses the uncertainties originating from the experimental data and reveals errors and ambiguities in the peak lists and potential instabilities of the downstream analysis.

Figure 2B illustrates the validation procedure for two CD79a samples with concentrations of 60 µM and 30 µM. The assignment for the 60 µM sample (blue bars) is stable and unique, although for some resampling trials not all residues were assigned. Notably worse results for the 30 µM sample (red bars) is explained by the lower signal-to-noise ratio and lack of the least sensitive (HN)CO(CA)NH experiment. A reduced probability of the major assignment and a significant fraction of the alternative assignment (Figure 2B, shaded and solid red bars, respectively) for residues 18–42 indicate that results for this region are highly suspicious. Detailed analysis shows that for the 30 µM sample the most probable assignments for residues E18, Y19, M35, and R41 are wrong, and the correct solution is found in the alternative assignment. This should be compared with the traditional approach, where the automated assignment is performed on a single set of peaks observed in the spectra processed using all acquired data. For this input, residues E20, N23 are not assigned and residues E18, Y19, E22, M35, Y36, E37, R41 are assigned incorrectly, while the assignment validation suite from the AutoAssign package [26] only reports suspicious assignment for residues D21, E22, G42 and not unique mapping for residues 57–62. Thus, the new resampling-based validation procedure better reveals residues with problematic assignments and more efficiently finds the correct solution.

Table 1 and Figure S1 summarize samples and *de novo* backbone assignments obtained using the presented CFPS-TA approach. These include five out of six TCR and BCR cytosolic domains, as well as mutated and modified forms of the domains. The experiments were performed in “native” (phosphate buffer), denaturing (6 M urea), and helix-promoting (20% trifluoroethanol, TFE) conditions. Depending on the sample, the TA measurement time required for the backbone assignment varied from 0.8 to 47 hours.

**Table 1:** Samples produced using CFPS and backbone assignments obtained with the automated TA procedure.

Sample	Mw kDa	Time^1^ hours	C µM	BMRB entry
CD79a	7.0	0.8	330	18867
CD79a	7.0	1.8	120	
CD79a	7.0	4.4	60	
CD79a	7.0	25.2	30	
CD79a+Urea	7.0	4.0	360^2^	18867
CD79a+TFE	7.0	46.5	40	
CD79a Y25E/Y36E	7.0	10.9	150^2^	
CD79a K4C/C33S	7.0	9.9	200	
CD79a MTSL^3^	7.0	13.2	250	
CD79b	5.5	18.4	150^2^	18884
CD3ε	6.2	4.0	230	18889
CD3ε +Urea	6.2	12.3	100	18889
CD3ε +TFE	6.2	7.0	120	
Cd3γ	5.2	7.6	150^2^	18890
TCRζ 4	12.9	10.0	500	15409

1 Measurement times correspond to the TA point of 90% assignment completeness and do not include the duration of the HNCO experiment, which is run prior to the TA.

2 Experiments were performed in NMR tubes of reduced diameter 3 mm (2.5 mm for CD79a+Urea).

3 CD79a with reduced MTSL label attached to C33.

4 Values for TCRζ are taken from our previous publication [25].

The lowest protein concentration, for which assignment was obtained, is 30 µM. It should be noted that apart from the relatively low protein concentrations the assignments were obtained despite of low signal dispersion and strong peak overlap, which is typical for IDPs. The signal separation in the spectra of our samples was significantly lower than the average of the BMRB database (Figure S2) for the corresponding protein sizes. For TCRζ, the signal separation is among the lowest in BMRB.

**Figure 4:**
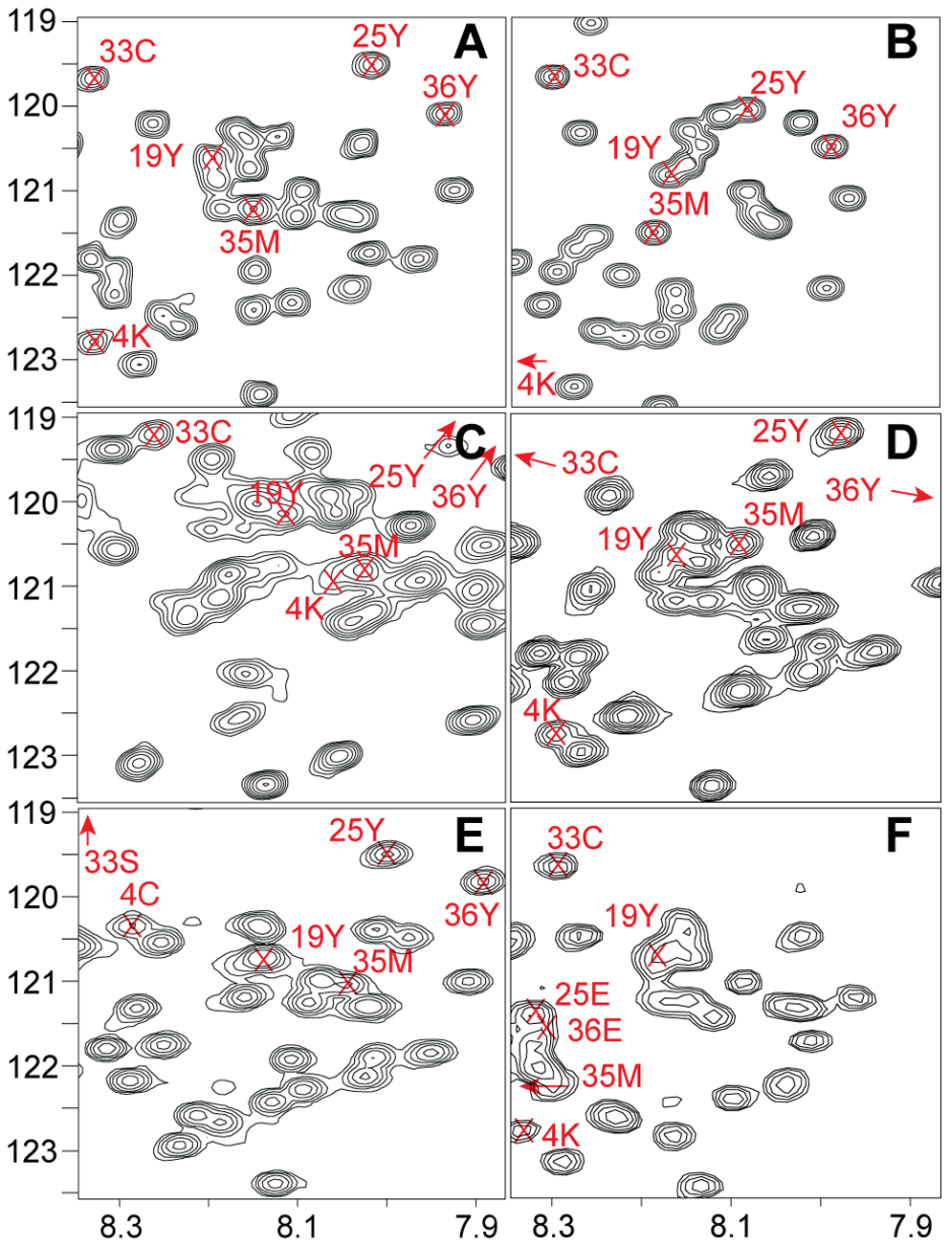
Zoomed region of the ^15^N-HSQC of CD79a in different conditions. (**A**) NaPi buffer, (**B**) 6 M urea, (**C**) 20% TFE, (**D**) reduced spin label (MTSL) attached to CD79a, (**E**) K4C/C35S form, (**F**) Y25E/Y36E form. Selected peaks are annotated to show rearrangements of the signal position for the different conditions (19Y, 35M) or position of the specific mutations (4K, 33C, 25Y, 36Y). Peaks outside the zoomed region are shown as arrows pointing towards the correct position.

**Figure 5:**
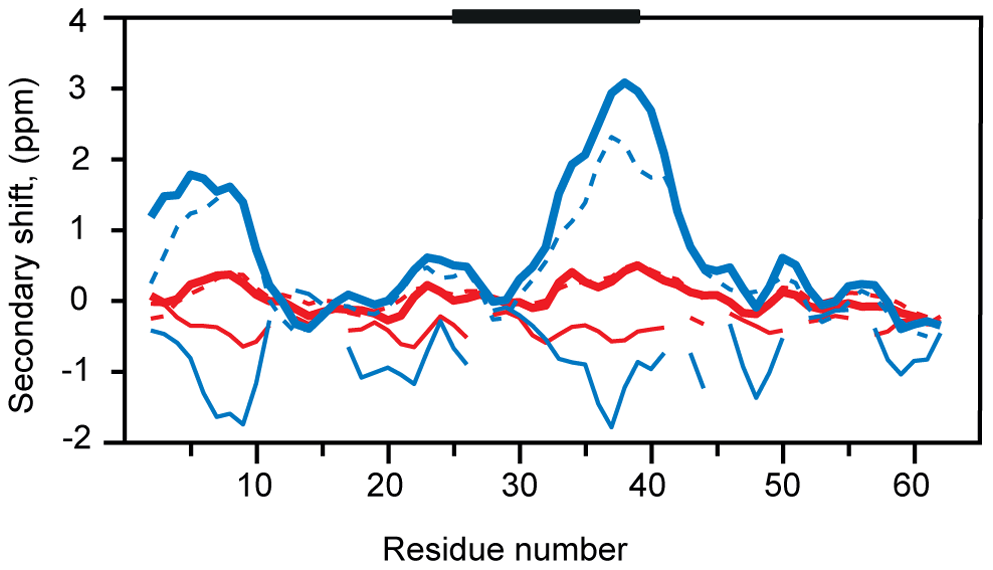
^13^C secondary chemical shifts for CD79a in native (red) and 20% TFE (blue) states. The values are obtained as the difference between the observed and random coil chemical shifts. The latter are measured in 6 M urea solution. The secondary chemical shifts of CO (dashed lines), CA (bold lines) and CB shifts (thin lines) clearly indicate α-helix propensities in the ITAM-motif region shown by horizontal black bars.

Figure 4 illustrates the usefulness of the approach for extensive characterization of a specific IDP. Independent *de novo* assignments for six CD79a samples were necessary due to rearrangement of the signal positions exacerbated by the strong peak overlap. The previously published [17] and our circular dichroism data (Figure S3) indicate that in the native condition the cytosolic domain of CD79a is mostly disordered. Analysis of the secondary chemical shifts has a potential to reveal regions of nascent structure in the protein [5], which is often relevant for IDP function. Figure 5 shows the ^13^C secondary chemical shifts of CD79a in native and TFE states referenced versus the so-called intrinsic random coil shifts [11] obtained in denatured condition of the protein. For CD79a in the native state, the CO, CA, and CB secondary chemical shifts shown in Figure 5 reveal small but significant propensity for α-helical structure around the ITAM sequence. An α-helix in this region was previously reported for CD79a interacting with Lyn protein-tyrosine kinase [33]. Addition of TFE to the experimental buffer is known to gradually enhance the α-helical population in regions with intrinsic helical preference. Adding TFE to the protein solution is thus often used to highlight the most α-helix-prone regions in the amino acid sequence [34]–[37]. The strongest onset of the helical structure upon addition of 20% TFE is observed around the ITAM (Figure 5), which indicates that this region has an underlying preference for α-helix formation coded in the amino acid sequence of CD79a. An additional residual helix is observed (Figure 5) at the N-terminus of CD79a. Here, similar to the ITAM region, relatively small but unambiguous α-helical secondary shifts are observed in the native state and are notably enhanced by the TFE. In this work, we show for the first time residual helical structure in the free state of CD79a cytosolic domain.

### Conclusions

Herein, we have presented an efficient methodology for extensive experimental characterization of highly disordered proteins in solution. Rapid and economical sample preparation and backbone assignment has been demonstrated for several TCR and BCR cytosolic domains. The technique allows chemical shifts perturbation analysis in multitude of IDP samples including site-specific mutants, chemical modification, different solution compositions, and in the presence of molecular interactions. The approach is demonstrated for five 6–13 kDa cytosolic domains of T- and B-cell receptors. In favorable cases, the three-dimensional BEST-TROSY spectra provide sufficient resolution for assigning IDPs up to 30 kDa [38]. Dealing with even larger systems should be possible by using NMR experiments of higher dimensionality and/or carbon and Hα detection [39], [40].

## Materials and Methods

### Chemicals and Bacterial Strains

All chemicals were from Sigma-Aldrich except where stated otherwise. TOP10 and BL21(DE3)-Rosetta 2 cells were from Life Technologies and Merck, respectively. ^13^C/^15^N labeled amino acids were from CIL.

### Cloning

Constructs for the human ITAM-containing cytosolic subunits were cloned. The cDNAs for CD79a (61 AA, P11912, 7.0 kDa), CD79b (49 AA, P40259, 5.5 kDa), CD3δ (45 AA, P04234, 5.1 kDa), CD3ε (57 AA, P07766, 6.2 kDa), CD3γ (44 AA, P09693 excluding the first Glycine, 5.2 kDa) and ξ (115 AA, P20963, 12.9 kDa) were purchased from Geneservice and subcloned into pEXP5-NT (Invitrogen), resulting in constructs with a 6xHis-tag fused to the N-terminus, followed by a TEV protease recognition site. The double mutant constructs of CD79a (K4C/C35S and Y25E/Y36E) were made by two rounds of QuickChange II XL Site-Directed Mutagenesis (Agilent Technologies). All clones were verified by sequencing. Cleavage with TEV protease resulted in 2 non-native residues (S and L) at the N terminus of the constructs. The vectors were transformed into One Shot® TOP10 chemically competent cells (Invitrogen). 100 ml cultures were grown overnight at 37°C, 200 rpm, and plasmids were purified with the Plus Maxi Kit (Qiagen).

### Expression and Purification

A batch format cell-free expression system based on an *E. coli* S12 extract was used for production of the peptides, as described [13]. The standard expression condition was 800 rpm for 2 hours at 30°C in 50 ml tubes on a Thermomixer Comfort (Eppendorf). For production of NMR samples, the cell-free expression reactions contained 1 mg/mL of mixture of ^15^N/^13^C labelled amino acids. 7 ml of the reaction was sufficient for producing 0.4 mg of pure CD79a/CD3ε. The identity and integrity of all expressed proteins have been confirmed using either mass spectrometry or NMR assignment. The cell-free synthesized human cytosolic subunits were purified for further analysis. The lysate from the centrifugation step was separated from the pellet and mixed 1∶2 with 20 mM Tris buffer pH 8.1, 8 M urea for 10 minutes. Ni-NTA resin (Qiagen) was equilibrated using buffer A (25 mM NaPi, 150 mM NaCl, 6 M urea, pH 7.2) and mixed with the protein-Tris buffer solution. Incubation at 4°C for 1.5 hours was followed by gravity flow purification with extensive washing using buffer A and buffer B (25 mM NaPi, 150 mM NaCl, 5 mM imidazole, pH 7.2). For protein elution, one-hour incubation with four times the gel column volume of buffer A (with 250 mM imidazole) was used. Imidazole was removed with PD10 desalting columns (GE Healthcare). The protein samples were mixed (10∶1) with his-tagged TEV protease prepared as described [41]. Cleavage was performed in a Thermomixer (Eppendorf) at 300 rpm, 23°C for 8 hours followed by 4°C for another 8 hours (CD79a, CD79b) or 23°C overnight (CD3ε, CD3γ, TCRζ). The TEV protease was separated from the cleaved protein using Ni-NTA resin as described above. The flow-through, containing the purified cleaved protein, was collected and filtered through a 0.2 µm syringe filter and loaded onto a RESOURCE RPC 3 ml column (Amersham Bioscience) connected to an FPLC system (ÄKTA Prime). Elution using a gradient of buffer C (0.065% trifluoroacetic acid (TFA), 2% acetonitrile) and buffer D (0.05% TFA, 80% acetonitrile) to 100% buffer D was performed and fractions containing protein of interest were lyophilized and stored at −20°C before use. To verify purity of the protein, SimplyBlue Coomassie (Invitrogen) staining of NuPAGE Novex 12% Bis-Tris Mini Gels (Invitrogen) was performed. PageRuler Prestained Protein Ladder (Fermentas) was used as a standard.

### NMR Sample Preparation

Lyophilized fractions were dissolved in NMR buffer (20 mM NaPi, pH 6.7, 1 mM EDTA, 1x Complete EDTA-free (Roche), 7–15% D_2_O and 2 mM DTT for cysteine-containing IDPs). For attachment of MTSL (1-oxy-2,2,5,5-tetramethyl-d-pyrroline-3-methyl)-methanethiosulfonate (Toronto Research Chemicals, Toronto) the NMR samples were treated with 10 mM DTT. A PD10 desalting column (GE Healthcare) was used to remove DTT and to change buffer to 10 mM NaPi, pH 7.0. 3–5 fold molar excess of MTSL dissolved in acetone was added and incubated overnight at 4°C. Unreacted MTSL was removed with a PD10 column followed by lyophilization of the sample. Reduced MTSL was obtained by adding 2 mM ascorbic acid to the solution.

### NMR Experiments

Tables S1 and S2 show details of the three-dimensional BETS-TROSY HNCO, HNCA, HN(CO)CA, HNCACB, HN(CO)CACB, (HN)CO(CA)NH experiments [38]. NMR experiments were performed at 25°C on Agilent spectrometers with Larmor frequencies of 800 MHz (CD79a+TFE and CD79a K4C/C33S) and 600 MHz (other samples) equipped with a cryo- (600 MHz) and room-temperature (800 MHz) pulse-field gradient triple-resonance probes. For CD79a+TFE, CD79a+MTSL, CD3ε, CD3ε+Urea, CD3ε+TFE samples, spectra were recorded using gradient sensitivity-enhanced pulse sequences from the BioPack library (Agilent Inc.); for the rest of the samples spectra were recorded using BEST-TROSY pulse sequences [14]. Acquisition time for ^15^N chemical shift evolution was set to the corresponding constant time period for the ^13^C-^15^N magnetization transfer; acquisition times for ^13^Cα/^13^Cβ and^ 13^CO evolution were set to 12 ms and 20 ms, respectively.

The NUS schedules were prepared by program *nussampler* from MDDNMR software package [15]. The sampling probability density was biased for ^13^Cα and ^13^Cβ evolution according to the single-bond ^13^C -^13^C homonuclear coupling constant of 35 Hz. The bias for the ^13^CO spectral dimension was set according to assumed transverse relaxation time of 20 ms.

### Secondary Chemical Shifts

Secondary chemical shifts (SCS) for ^13^Cα, ^13^Cβ and ^13^CO resonances were calculated by subtracting intrinsic random coil chemical shifts [11], [42], which were measured for the protein in 6 M urea, from the corresponding values obtained for “native” and “20% TFE” states. For presentation in Figure 5, the values were smoothed by three-residue running average.

### Targeted Acquisition

TANSY (Targeted Acquisition NMR Spectroscopy) procedure, which we introduce in this work, combines several techniques to achieve high efficiency of data collection and analysis. For the backbone assignment, it uses a set of fast-pulsing triple resonance BEST-TROSY experiments [14], [38] featuring reduced measurement times and improved sensitivity and resolution for protein samples. The spectra are acquired using non-uniform sampling (NUS) with the sampling schedule tailored to the signal intensity for improving sensitivity [15]. In the sampling limiting case, NUS gives dramatic savings of measurement time because only a small fraction of the traditional full data set is collected (Table S2). Additional time saving is obtained in the course of the TA [43] by optimizing time allocations for the individual experiments and duration of the whole set. Processing of the NUS spectra is performed using recursive co-MDD [25], [43], which allows high quality spectra reconstruction from a very small number of measurements. Further time saving is obtained by replacing lengthy manual work with the spectra by the automated hyper-dimensional signal detection [25] and automated backbone assignment [26].

TANSY software consists of two separate modules: the acquisition module (AM) and the processing and analysis module (PAM). The former is running on an NMR spectrometer and organizes the stepwise incremental data collection. AM is implemented using macro and graphical interface features of VnmrJ (Agilent Technologies, Inc.) and TopSpin (Bruker Biospin) spectrometer software. AM runs a set of NMR experiments using a NUS schedule. The acquired data are continuously saved to computer disk and become immediately available for the downstream processing by PAM. The PAM can operate in real-time with the AM or can be used as an off-line processing and analysis tool. PAM is implemented in the Python programming language (http://python.org/) with QT (http://qt.nokia.com/) based graphical interface. PAM uses MDDNMR [15] and NMRPipe [44] programs for the spectra processing, its own routines for signal detection in one-dimensional MDD shapes, and AutoAssign [26] for the automated backbone assignment. The output of the PAM module comprises a set of processed spectra in an NMRPipe format, peak lists, assignment tables, and a set of progress and quality scores. The output can be easily imported into various programs, including CCPN Analysis [45] and CARA (http://cara.nmr.ch/) for manual inspection of the assignment and further analysis. TANSY software is available from the authors upon request.

### Spectral Acquisition

The acquisition module introduced in the previous section performs iterative incremental acquisition of NUS experimental sets [46]. The most sensitive experiment from the set, HNCO is recorded first using NUS with 10–30% sparse sampling. Subsequently, at each TA step, every experiment from the suite has a short run over a small fraction (e.g. 0.5–1.0%) of points from the NUS schedule. The number of points differs for individual experiments and can be adjusted during the course of the TA procedure in order to balance the experiment time allocations. Thus, AM performs incremental acquisition for all experiments in an interleaved manner and provides input amenable for the real-time analysis by PAM.

### Processing and Analysis Module

First, the HNCO experiment is processed with recursive MDD [47] or compressed sensing [48]. A 3D peak list is produced with program *pkFindROI* from the NMRPipe package [44]. At this stage, the peak list can be manually verified to add missing and remove occasional false peaks. Although ideally MDD components have a one-to-one relation to peaks in the spectrum, in practice it is not always so. In the case of mixing [49], one component may describe several peaks. Some of the components may correspond to spectral artifacts or strong noise features. “Clean” MDD components, i.e. those unambiguously related to peaks, are obtained by fixing ^15^N and ^13^CO frequencies to the values in the peak list. Namely, time domain shapes for the constant time ^15^N dimension are analytically calculated as 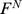 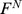 = *cos(ω_N_ t)+I sin(ω_N_ t).* The ^13^CO time domain shapes are presented as products of known frequency 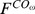 = *cos(ω_CO_ t)+I sin(ω_CO_ t)* and unknown decay 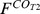 parts. Thus, the standard MDD model 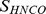 of the HNCO spectrum:
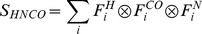
(1)where summation index *i* runs over all components and the symbol ∶ denotes the tensor product operation is replaced by:



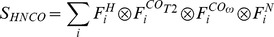
(2)Here 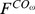 and 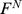 are known from the peak list and only the amide proton 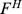 and carbonyl decay 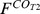 shapes have to be found in the decomposition. Furthermore, 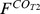 shapes are assumed to represent exponential decays, for which recursive MDD can be applied. After performing the frequency-restrained MDD decomposition (Eq. 2), the clean components are obtained. The 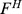 and 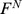 shapes from the clean components are fixed in the subsequent processing of other experiments, so that only the remaining ^13^C shapes are defined in the co-MDD and all peaks are naturally grouped to spin systems. Straightforward and robust signal identification in 1D shapes produces only a few, if any, false peaks. At each TA step, the set of peak lists serves as an input for the automated sequence-specific assignment, which is performed by program AutoAssign [26]. Thus, both the peak counts and the assignment progress can be monitored in real time. This allows adjustment of measurement time for the individual experiments and indicates when the TA procedure can be finished.

### Validation using Jackknife-resampling

**Figure 6:**
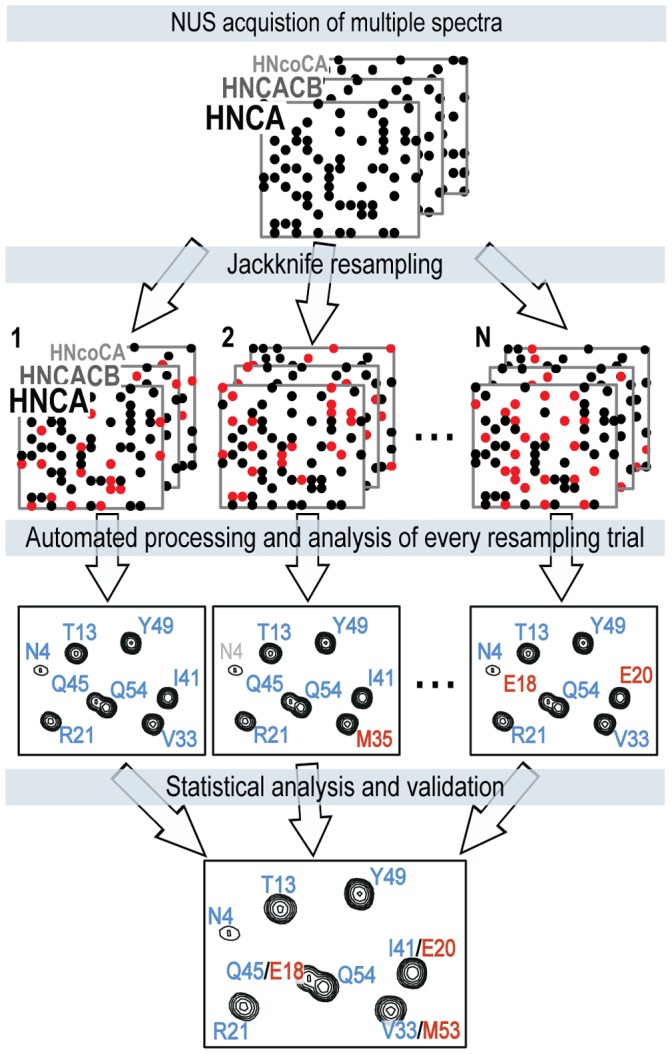
Assignment validation procedure with jackknife resampling. See text for explanations.

In our jackknife procedure, a set of realizations is produced for every peak position and atom assignment. This provides necessary statistics for the calculation of the probability scores for the primary and alternative assignments, as shown in Figure 2B. The procedure is illustrated for a hypothetical spectrum shown in in Figure 6. Specifically, N = 30 resampling trials are produced for every triple resonance experiment (i.e. HNCA, HNCACB, etc) by randomly omitting 30% of data points. For the so-called deleted jackknife procedure [31] used in this work, the amount of the omitted data should exceed the square root of the total number of data points. The omission, however, must not significantly reduce sensitivity of the spectra and the chances for peak identification. Our choice for omitting 30% data fulfills both requirements. In Figure 6, the omitted points are shown in red. For every trial, the spectra are processed and the corresponding analysis including peak detection and automatic assignment is performed. Due to different realization of noise and spectral artifacts in the resampled spectra, the peak lists and assignments may vary from one trial to another. For example, in the second trial shown in the Figure 6, residue N4 (gray) was not assigned due to low intensities of the corresponding peaks. Furthermore, the spin system marked in red was assigned to residue M35, which differs from the assignment found in most of the other trials. The assignment probability score for a particular residue is calculated as a fraction of trials where this assignment is obtained. The alternative assignments are ranked based in their probability scores and the two most probable are reported in Figure 2A.

## Supporting Information

Figure S1 **Assignment annotated 1H-15N projections from native 3D HNCO spectra.** (A) CD79a, (B) CD79b, (C) CD3ε, (D) CD3γ. Crowded regions are shown as inserts.
(EPS)

Figure S2
**Peak separation index (PSI) versus protein size.** PSI is calculated as a sum of ten minimal pairwise distances in 2D ^1^H-^15^N plane between assigned amide groups for each protein: 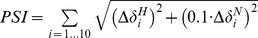, where Δ*δ^H^* and Δ*δ^N^* are ppm distances in amide proton and nitrogen dimensions, respectively. **(**yellow**)** PSIs of 10% of randomly chosen proteins from full BMRB database; **(**blue**)** 65 protein entries found in the IDP subset of BMRB; **(**red**)** twelve TCR and BCR cytosolic domains constructs: (1) CD3γ; (2) CD79b; (3) CD3ε, 20% TFE; (4) CD3ε in 6 M urea; (5) CD3ε; (6) CD79a TFE 20%, (7) CD79a and CD79a in 6 M Urea; (8) CD79a MTSL; (9) CD79a Y25E/Y36E and K4C/C33S; (10) TCRζ.(EPS)

Figure S3 **CD spectra of CD79a samples.** Far-UV circular dichroism spectra (190–250 nm) were recorded at 25°C on a ChiraScan CD spectrometer (Applied Photophysics) with 0.38 mg/ml CD79a using a 0.1 cm path-length quartz cuvette. 5 mM NaPi pH 6.7 buffer with 0.5 mM DTT was used for sample dilution. The sample blank was subtracted and 5 scans averaged for the protein. **(**thick line**)** CD spectra for CD79a in buffer, **(**thin line**)** 10% trifluoroethanol (TFE), **(**grey line**)** 20% TFE and **(**dashed line**)** 30% TFE. Under native condition, CD79a shows a characteristic random coil curve with the minimum around 200 nm. As TFE concentration increases, the CD spectra gradually adopt alpha helical features (i.e. minima at 222 nm, 208 nm and a positive value at 190 nm). The presented CD spectrum for the native condition is similar to the spectrum reported in [17].
(EPS)

Table S1 **The NMR experimental parameters for backbone assignments.**
(PDF)

Table S2 **Measurement times (min) needed for reaching 90% completeness of the sequential backbone assignment for CD79a samples at four different protein concentrations.**
(PDF)

## References

[1] Dyson HJ, Wright PE (2005) Intrinsically unstructured proteins and their functions. Nat Rev Mol Cell Biol 6: 197–208. 15738986 10.1038/nrm1589

[2] Tompa P (2010) Structure and function of intrinscially disordered proteins. Boca Raton: Chapman & Hall/CRC Press. xxvii, 331 p. p.

[3] Uversky VN, Dunker AK (2010) Understanding protein non-folding. Biochim Biophys Acta 1804: 1231–1264. 20117254 10.1016/j.bbapap.2010.01.017 PMC2882790

[4] Stein A, Pache RA, Bernado P, Pons M, Aloy P (2009) Dynamic interactions of proteins in complex networks: a more structured view. Febs J 276: 5390–5405. 19712106 10.1111/j.1742-4658.2009.07251.x

[5] Mittag T, Forman-Kay JD (2007) Atomic-level characterization of disordered protein ensembles. Curr Opin Struct Biol 17: 3–14. 17250999 10.1016/j.sbi.2007.01.009

[6] Hiller S, Garces RG, Malia TJ, Orekhov VY, Colombini M, et al (2008) Solution structure of the integral human membrane protein VDAC-1 in detergent micelles. Science 321: 1206–1210. 18755977 10.1126/science.1161302 PMC2579273

[7] Tugarinov V, Kay LE (2005) Methyl groups as probes of structure and dynamics in NMR studies of high-molecular-weight proteins. ChemBioChem 6: 1567–+. 10.1002/cbic.200500110 16075427

[8] Tzakos AG, Grace CRR, Lukavsky PJ, Riek R (2006) NMR techniques for very large proteins and RNAs in solution. Annual Review of Biophysics and Biomolecular Structure. Palo Alto: Annual Reviews. pp. 319–342. 10.1146/annurev.biophys.35.040405.102034 16689639

[9] Motackova V, Novacek J, Zawadzka-Kazimierczuk A, Kazimierczuk K, Zidek L, et al (2010) Strategy for complete NMR assignment of disordered proteins with highly repetitive sequences based on resolution-enhanced 5D experiments. J Biomol NMR 48: 169–177. 20890634 10.1007/s10858-010-9447-3 PMC2966349

[10] Narayanan RL, Durr UHN, Bibow S, Biernat J, Mandelkow E, et al (2010) Automatic Assignment of the Intrinsically Disordered Protein Tau with 441-Residues. J Am Chem Soc 132: 11906–11907. 20687558 10.1021/ja105657f

[11] Kjaergaard M, Poulsen FM (2012) Disordered proteins studied by chemical shifts. Prog Nucl Magn Reson Spectrosc 60: 42–51. 22293398 10.1016/j.pnmrs.2011.10.001

[12] Salmon L, Nodet G, Ozenne V, Yin GW, Jensen MR, et al (2010) NMR Characterization of Long-Range Order in Intrinsically Disordered Proteins. J Am Chem Soc 132: 8407–8418. 20499903 10.1021/ja101645g

[13] Pedersen A, Hellberg K, Enberg J, Karlsson BG (2011) Rational improvement of cell-free protein synthesis. N Biotechnol 28: 218–224. 20603235 10.1016/j.nbt.2010.06.015

[14] Favier A, Brutscher B (2011) Recovering lost magnetization: polarization enhancement in biomolecular NMR. J Biomol NMR 49: 9–15. 21190063 10.1007/s10858-010-9461-5

[15] Orekhov VY, Jaravine VA (2011) Analysis of non-uniformly sampled spectra with Multi-­Dimensional Decomposition. Prog Nucl Magn Reson Spectrosc 59: 271–292. 21920222 10.1016/j.pnmrs.2011.02.002

[16] Sigalov A, Aivazian D, Stern L (2004) Homooligomerization of the cytoplasmic domain of the T cell receptor xi chain and of other proteins containing the immunoreceptor tyrosine-based activation motif. Biochemistry 43: 2049–2061. 14967045 10.1021/bi035900h

[17] Sigalov AB, Aivazian DA, Uversky VN, Stern LJ (2006) Lipid-binding activity of intrinsically unstructured cytoplasmic domains of multichain immune recognition receptor signaling subunits. Biochemistry 45: 15731–15739. 17176095 10.1021/bi061108f PMC2528957

[18] Kigawa T, Yabuki T, Yoshida Y, Tsutsui M, Ito Y, et al (1999) Cell-free production and stable-isotope labeling of milligram quantities of proteins. FEBS Lett 442: 15–19. 9923595 10.1016/s0014-5793(98)01620-2

[19] Ozawa K, Headlam MJ, Schaeffer PM, Henderson BR, Dixon NE, et al (2004) Optimization of an Escherichia coli system for cell-free synthesis of selectively N-15-labelled proteins for rapid analysis by NMR spectroscopy. Eur J Biochem 271: 4084–4093. 15479237 10.1111/j.1432-1033.2004.04346.x

[20] Vinarov DA, Lytle BL, Peterson FC, Tyler EM, Volkman BF, et al (2004) Cell-free protein production and labeling protocol for NMR-based structural proteomics. Nature Methods 1: 149–153. 15782178 10.1038/nmeth716

[21] Klammt C, Lohr F, Schafer B, Haase W, Dotsch V, et al (2004) High level cell-free expression and specific labeling of integral membrane proteins. Eur J Biochem 271: 568–580. 14728684 10.1111/j.1432-1033.2003.03959.x

[22] Kanter G, Yang JH, Voloshin A, Levy S, Swartz JR, et al (2007) Cell-free production of scFv fusion proteins: an efficient approach for personalized lymphoma vaccines. Blood 109: 3393–3399. 17164345 10.1182/blood-2006-07-030593 PMC1852255

[23] Woodrow KA, Airen IO, Swartz JR (2006) Rapid expression of functional genomic libraries. J Proteome Res 5: 3288–3300. 17137330 10.1021/pr050459y

[24] Kurotani A, Takagi T, Toyama M, Shirouzu M, Yokoyama S, et al (2010) Comprehensive bioinformatics analysis of cell-free protein synthesis: identification of multiple protein properties that correlate with successful expression. Faseb J 24: 1095–1104. 19940260 10.1096/fj.09-139527

[25] Jaravine V, Zhuravleva A, Permi P, Ibraghimov I, Orekhov VY (2008) Hyper-dimensional NMR spectroscopy with nonlinear sampling. J Am Chem Soc 130: 3927–3936. 18311971 10.1021/ja077282o

[26] Moseley HNB, Sahota G, Montelione GT (2004) Assignment validation software suite for the evaluation and presentation of protein resonance assignment data. J Biomol NMR 28: 341–355. 14872126 10.1023/B:JNMR.0000015420.44364.06

[27] Brünger AT, Clore GM, Gronenborn AM, Saffrich R, Nilges M (1993) Assessing the quality of solution nuclear magnetic resonance structures by complete cross-validation. Science 261: 328–331. 8332897 10.1126/science.8332897

[28] Wang BW, Wang YJ, Wishart DS (2010) A probabilistic approach for validating protein NMR chemical shift assignments. J Biomol NMR 47: 85–99. 20446018 10.1007/s10858-010-9407-y

[29] Bagaria A, Jaravine V, Huang YPJ, Montelione GT, Guntert P (2012) Protein structure validation by generalized linear model root-mean-square deviation prediction. Protein Sci 21: 229–238. 22113924 10.1002/pro.2007 PMC3324767

[30] Efron B (1982) The jackknife, the bootstrap, and other resampling plans. Philadelphia, Pa.: Society for Industrial and Applied Mathematics. vii, 92 p. p.

[31] Efron B, Tibshirani R (1993) An introduction to the bootstrap. New York: Chapman & Hall. xvi, 436 p. p.

[32] Zhang HY, Neal S, Wishart DS (2003) RefDB: A database of uniformly referenced protein chemical shifts. J Biomol NMR 25: 173–195. 12652131 10.1023/a:1022836027055

[33] Gaul BS, Harrison ML, Geahlen RL, Burton RK, Post CB (2000) Substrate recognition by the Lyn protein-tyrosine kinase - NMR structure of the immunoreceptor tyrosine-based activation motif signaling region of the B cell antigen receptor. J Biol Chem 275: 16174–16182. 10748115 10.1074/jbc.M909044199

[34] Buck M (1998) Trifluoroethanol and colleagues: cosolvents come of age. Recent studies with peptides and proteins. Q Rev Biophys 31: 297–355. 10384688 10.1017/s003358359800345x

[35] Dyson HJ, Merutka G, Waltho JP, Lerner RA, Wright PE (1992) Folding of peptide-fragments comprising the complete sequence of proteins - models for initiation of protein folding.1. Myohemerythrin. J Mol Biol 226: 795–817. 1507227 10.1016/0022-2836(92)90633-u

[36] Dyson HJ, Sayre JR, Merutka G, Shin HC, Lerner RA, et al (1992) Folding of peptide-fragments comprising the complete sequence of proteins - models for initiation of protein folding.2. Plastocyanin. J Mol Biol 226: 819–835. 1507228 10.1016/0022-2836(92)90634-v

[37] Segawa S, Fukuno T, Fujiwara K, Noda Y (1991) Local structures in unfolded lysozyme and correlation with secondary structures in the native conformation - helix-forming or helix-breaking propensity of peptide segments. Biopolymers 31: 497–509. 1868165 10.1002/bip.360310505

[38] Solyom Z, Schwarten M, Geist L, Konrat R, Willbold D, et al. (2013) BEST-TROSY experiments for time-efficient sequential resonance assignment of large disordered proteins. J Biomol NMR DOI 10.1007/s10858-013-9715-0: in press. 10.1007/s10858-013-9715-0 23435576

[39] Bermel W, Bertini I, Felli IC, Lee YM, Luchinat C, et al (2006) Protonless NMR experiments for sequence-specific assignment of backbone nuclei in unfolded proteins. J Am Chem Soc 128: 3918–3919. 16551093 10.1021/ja0582206

[40] Mantylahti S, Aitio O, Hellman M, Permi P (2010) HA-detected experiments for the backbone assignment of intrinsically disordered proteins. J Biomol NMR 47: 171–181. 20437194 10.1007/s10858-010-9421-0

[41] van den Berg S, Lofdahl PA, Hard T, Berglund H (2006) Improved solubility of TEV protease by directed evolution. J Biotechnol 121: 291–298. 16150509 10.1016/j.jbiotec.2005.08.006

[42] Modig K, Jürgensen VW, Lindorff-Larsen K, Fieber W, Bohr HG, et al (2007) Detection of initiation sites in protein folding of the four helix bundle ACBP by chemical shift analysis. FEBS Lett 581: 4965–4971. 17910956 10.1016/j.febslet.2007.09.027

[43] Jaravine V, Orekhov VY (2006) Targeted Acquisition for Real-Time NMR Spectroscopy. J Am Chem Soc 128: 13421–13426. 17031954 10.1021/ja062146p

[44] Delaglio F, Grzesiek S, Vuister GW, Zhu G, Pfeifer J, et al (1995) NMRPipe: a multidimensional spectral processing system based on UNIX pipes. J Biomol NMR 6: 277–293. 8520220 10.1007/BF00197809

[45] Vranken WF, Boucher W, Stevens TJ, Fogh RH, Pajon A, et al (2005) The CCPN data model for NMR spectroscopy: development of a software pipeline. Proteins 59: 687–696. 15815974 10.1002/prot.20449

[46] Jaravine VA, Orekhov VY (2006) Targeted acquisition for real-time NMR spectroscopy. J Am Chem Soc 128: 13421–13426. 17031954 10.1021/ja062146p

[47] Jaravine V, Ibraghimov I, Orekhov VY (2006) Removal of time barrier for high-resolution multidimensional NMR spectroscopy. Nature Methods 3: 605–607. 16862134 10.1038/nmeth900

[48] Kazimierczuk K, Orekhov VY (2011) Accelerated NMR Spectroscopy by Using Compressed Sensing. Angew Chem-Int Edit 50: 5556–5559. 10.1002/anie.201100370 21538743

[49] Orekhov VY, Ibraghimov IV, Billeter M (2001) MUNIN: A new approach to multi-dimensional NMR spectra interpretation. J Biomol NMR 20: 49–60. 11430755 10.1023/a:1011234126930

