# Supplementary material for: Highly Efficient NMR Assignment of Intrinsically Disordered Proteins: Application to B- and T Cell Receptor Domains
Source: PLoS One. 2013 May 7;8(5):e62947. doi: 10.1371/journal.pone.0062947 (PMC3647075; doi:10.1371/journal.pone.0062947)
Supplement: Table S1 — The NMR experimental parameters for backbone assignments. (PDF) [file pone.0062947.s004.pdf]

Table S1. The NMR experimental parameters for backbone assignments.

Sample	C µM	Exp. Type[1]	Relaxation delay, sec	Number of transients[2]	Number of FIDs[2]
CD79a	330	BT	0.1	4, 4, 4, 4, 4	32,16,64,32,32
CD79a	120	BT	0.1	4, 4, 4, 4, 4	32,16,64,32,32
CD79a	60	BT	0.1	4, 4, 4, 4, 4	32,16,64,32,32
CD79a	30	BT	0.1	32, 16, 32, 32, -[3]	32,16,64,32, -[3]
CD79a+Urea	360[4]	BT	0.1	16, 8, 32, 16, 16	32,16,32,32,32
CD79a+TFE	45	Bp[5]	1.0	4, 4, 4, 4, 8	64,32,64,128,64
CD79a Y25E/Y36E	150[4]	BT	0.2	16, 8, 32, 16, 32	13,16,36,43,13
CD79a K4C/C33S	200	BT[5]	0.2	16, 8, 32, 32, 32	32,32,32,32,32
CD79a MTSL	250	Bp	1.0	4, 4, 4, 4, 8	32,16,64,64,32
CD79b	150[4]	BT	0.2	16, 8, 32, 16, 32	16,19,43,52,16
CD3e	230	Bp	1.0	4, 4, 4, 4, 8	32,16,64,64,32
CD3e+Urea	100	Bp	1.2	4, 4, 4, 4, 8	32,16,64,64,32
CD3e+TFE	120	Bp	1.2	4, 4, 4, 4, 8	32,16,64,32,32
CD3g	150[2]	BT	0.2	16, 8, 32, 16, 64	13,16,36,43,13

[1]Experimental library: BT – BEST-TROSY, Bp – BioPack. [2]Number of transients and hyper-complex FIDs per one TA step are given for HNCA, HN(CO)CA, HNCACB, HN(CO)CACB, (HN)CO(CA)NH experiments, respectively. [3]The least sensitive (HN)CO(CA)NH experiment was excluded for the 30 µM sample. [4] These experiments were performed in NMR tubes of reduced diameter 3 mm (2.5 mm for CD79a+Urea). [5]These experiments were performed on a 800MHz magnet equipped with room temperature probe, which has ca 1.5 times lower sensitivity in comparison with the 600 MHz cold probe used for the other samples.
