# Supplementary material for: Highly Efficient NMR Assignment of Intrinsically Disordered Proteins: Application to B- and T Cell Receptor Domains
Source: PLoS One. 2013 May 7;8(5):e62947. doi: 10.1371/journal.pone.0062947 (PMC3647075; doi:10.1371/journal.pone.0062947)
Supplement: Table S2 — Measurement times (min) needed for reaching 90% completeness of the sequential backbone assignment for CD79a samples at four different protein concentrations. (PDF) [file pone.0062947.s005.pdf]

Table S2. Measurement times (min) needed for reaching 90% completeness of the sequential backbone assignment for CD79a samples at four different protein concentrations. Values in parenthesis denote the corresponding sparse levels (%) of data sampling for individual experiments.

Concentration / Experiment	330 μM	120 μM	60 μM	30 μM
HNCO [a]	90 (30)	90 (30)	90 (30)	360 (30)
HNCA	7.7 (5.9)	18 (8.3)	43.5 (20.1)	326 (19)
HN(CO)CA	4.5 (2.5)	10 (3.4)	24 (8.3)	90 (7.8)
HN(CA)CB	16.7 (6.3)	39 (8.8)	94.5 (21.2)	709 (20)
HN(COCA)CB	9 (1.6)	21 (3.6)	51.5 (8.8)	385 (8.3)
(HN)CO(CA)NH[b]	9 (3)	21 (7)	51.5 (17)	-
Total TA	47	109.8	265	1511

[a] The HNCO experiment was recorded prior to the TA procedure and therefore is not included in the total TA timing.

[b] The least sensitive (HN)CO(CA)NH experiment was not used for the 30 μM sample.
